# Eye Movements Provide an Index of Veridical Memory for Temporal Order

**DOI:** 10.1371/journal.pone.0125648

**Published:** 2015-05-20

**Authors:** Thanujeni Pathman, Simona Ghetti

**Affiliations:** 1 Department of Psychology, University of North Carolina at Greensboro, Greensboro, North Carolina, United States of America; 2 Department of Psychology, University of California at Davis, Davis, California, United States of America; CEA.DSV.I2BM.NeuroSpin, FRANCE

## Abstract

The present research examined whether eye movements during retrieval capture the relation between an event and its temporal attributes. In two experiments (N=76), we found converging evidence that eye movements reflected the veridicality of memory for temporal order seconds before overt memory judgments, suggesting that these movements captured indirect access to temporal information. These eye movements did not entirely depend on the amount of contextual cueing available (Experiment 1) and reflected the unique ordinal position of an event in a sequence (Experiment 2). Based on our results, we conclude that eye movements reflected the absolute temporal order of past events.

## Introduction

We all experience the fallacies of our memories. Though memory inaccuracy may be mildly aggravating, it frequently carries important consequences, such as when one misremembers taking a medication, or provides faulty eyewitness testimony in the courtroom. Some recent research has shown that eye movements can index memory accurately independent of explicit response or verbal reports ([1–3]; see also [4–9]; see [10] for review), holding the promise to be used even in populations that could form reliable memories, but are unable to provide detailed verbal accounts (e.g., young children, certain neurological patients) (for examples see [11–13]). Despite this promise, it is currently unknown whether eye movements can capture all relevant features of episodic memories (e.g., temporal, spatial features). If eye movements are to be proposed as a possible alternative or addition to verbal reports, it is critical to establish to what kind of information they respond over long delays. One such piece of information concerns the temporal order with which events occur.

Memory for temporal order is a central feature of episodic memory [14–17]. Practically, establishing the order of past events is critical in several situations including skipping a daily dose of a medication or placing an individual in a crime context at a damning time. In the present investigation, we examine whether and how eye movements can be used to study temporal long-term memory. Before discussing the present approach we briefly review how eye movements can be used to examine memory for the features of past events.

### Eye movements, Cognition and Memory: A Long History

Eye movement measures have long been used to investigate various cognitive processes (for reviews see [18–20]), including language [21], visual-spatial attention and object search [22], semantic memory and perception [23], working memory ([24], for review) and episodic memory ([10], for review). Some of these studies have focused on scene memory or spatial features of events, including inter- or intra-object spatial relations [25–32]. This work shows that eye movement patterns are influenced by memory for items bound in scenes. For example, altering portions of previously learned scenes alters viewing patterns [5, 9, 33, 34].

Eye movements may also facilitate retrieval of visual-spatial information. For example, Johansson and Johansson [29] showed that constraining eye movements during retrieval (by asking participants to fixate on a central fixation) caused longer reaction times in a task involving memory for inter-object spatial relations. Overall, these studies point to ways in which eye movement behavior is guided by memory representations [10]. Further, these studies suggest that eye movement measures may provide unique information about memory processes which might complement that obtained with overt measures. In the next section, we describe a line of research that has examined long-term episodic memory and binding of an event to a context, and guided the present investigation.

### Eye Movements and Memory for Item-Context Associations

Within this rich tradition, a line of research specifically focused on how eye movements may capture relational processes: binding elements of an episode together into an integrated episodic memory representation. In some eye movement studies of item-context binding, participants study faces superimposed on background scenes. At test, participants are presented with a previously studied background scene and are asked to select the matching face from a 3-face display, which contains the target face and two distracter faces, which were presented with other background scenes during study. Hannula, Cohen and colleagues found that participants spend a disproportionate amount of time viewing the correctly selected faces (correct trials) compared to incorrectly selected distracter faces (incorrect trials) early in the trial, before overt responses are made, suggesting eye movements reflect veridical memory. Only selected items are compared because previous research has shown that there is increased viewing to selected compared to non-selected items [35] across the trial length. Examination of eye movements toward the selected face for correct compared to incorrect trials avoids response selection confounds (see [36] for discussion). Eye movements are examined in discrete time bins across the trial length and researchers use this approach to focus on early eye movements, that precede well in time the moment when a decision response is rendered, based on the idea that early in the trial, we will be more likely to see episodic representations being reinstated [2,3, 11–13].

Researchers have used this approach to show that memory influences eye-movement patterns early, in advance of explicit recognition [2, 37] and the eye-movement patterns seem to be obligatory (i.e., occur soon after stimulus onset, even with no memory retrieval demand) [36]. As discussed by Kumaran and Wagner [38], eye-movement effects (disproportionate viewing to correctly selected target) [3] may contribute to the accumulation of evidence needed to make an explicit choice, and may “reflect early emerging, and perhaps relatively pure, signatures of memory retrieval…” (p. 563). In addition, these eye-movement effects are linked to hippocampal function based on studies involving amnesic patients with lesions to the hippocampus [2] and neuroimaging with typical adults [3]. Overall, this work offers powerful evidence that eye movements may provide a window into the integrity of episodic representations mediated by the hippocampus, independent of conscious episodic recollection [10].

Given that the hippocampus supports memory for temporal order [39] and the eye-movement effects discussed earlier [2], we use this approach to examine early eye movements in an item-temporal memory task, and predicted that early eye movements reflected veridical long-term memory for temporal order. In two experiments, we tested whether and how that is the case.

## Experiment 1

Within a single paradigm adapted from Pathman and Ghetti [40], we examined whether eye movements could provide an index of the precise temporal order of events (B happened immediately after A), and whether they additionally responded to the broader temporal context of an event (B happened around the same time as A). Precise order and context may be both organizing principles with which we retain temporal information about events of our past and both rely on the hippocampus [41,42]. While addressing the main question, we also manipulated the amount of contextual reinstatement during retrieval. Previous studies reporting eye-movement effects involved strong overlap between encoding and retrieval conditions [3]. Thus, we deemed it important to account for this factor.

Like in previous investigations using the face-scene paradigm [2,3,11] we examined proportion of looking to selected items for correct compared to incorrect trials to determine if there was evidence of similar “relational eye-movement effects” [38] for temporal memory. Following these previous investigations, we examined eye movements across the trial length in discrete time bins because, as discussed earlier, eye movements soon after stimulus onset may provide us with an indication of reinstatement of memory representations and covert processing that could contribute to overt choice.

### Participants

Thirty-seven young adults (*M* = 21.41 years, *SD* = 2.76; 56.8% females) took part in the study. All participants completed one session that was approximately 1.5 hours long, including breaks. The University of California, Davis Institutional Review Board approved the protocol. Participants were recruited from a university participant pool, provided written consent, and received course credit for their participation. One participant was excluded because of chance performance in the retrieval phase (across conditions, described below). We conducted convenience sampling with sample size comparable with previous eye movement investigations ([2]: 36 undergraduate students; [36]: 40 undergraduate students).

### Stimuli and Apparatus

We selected 350 items from a bank of standardized color photographs of objects [43]. Stimuli were presented on a white background using a Tobii T-120 Eye Tracker (Tobii.com; eye tracker integrated into a 17-inch monitor). Calibration procedures, conducted before each run of trials, consisted of participants following a red circle that moved to 5 different locations on the screen. Default Tobii fixation filter settings (velocity threshold: 35 pixels/samples; distance threshold: 35 pixels) were used for eye movement data reduction. Tobii guidelines [44] were used to test the timing of our specific eye-tracker/computer setup and the synchronization offset was on average 65.22 ms (*SD* = 17.12).

### Procedure

#### Encoding phase

**Fig 1:**
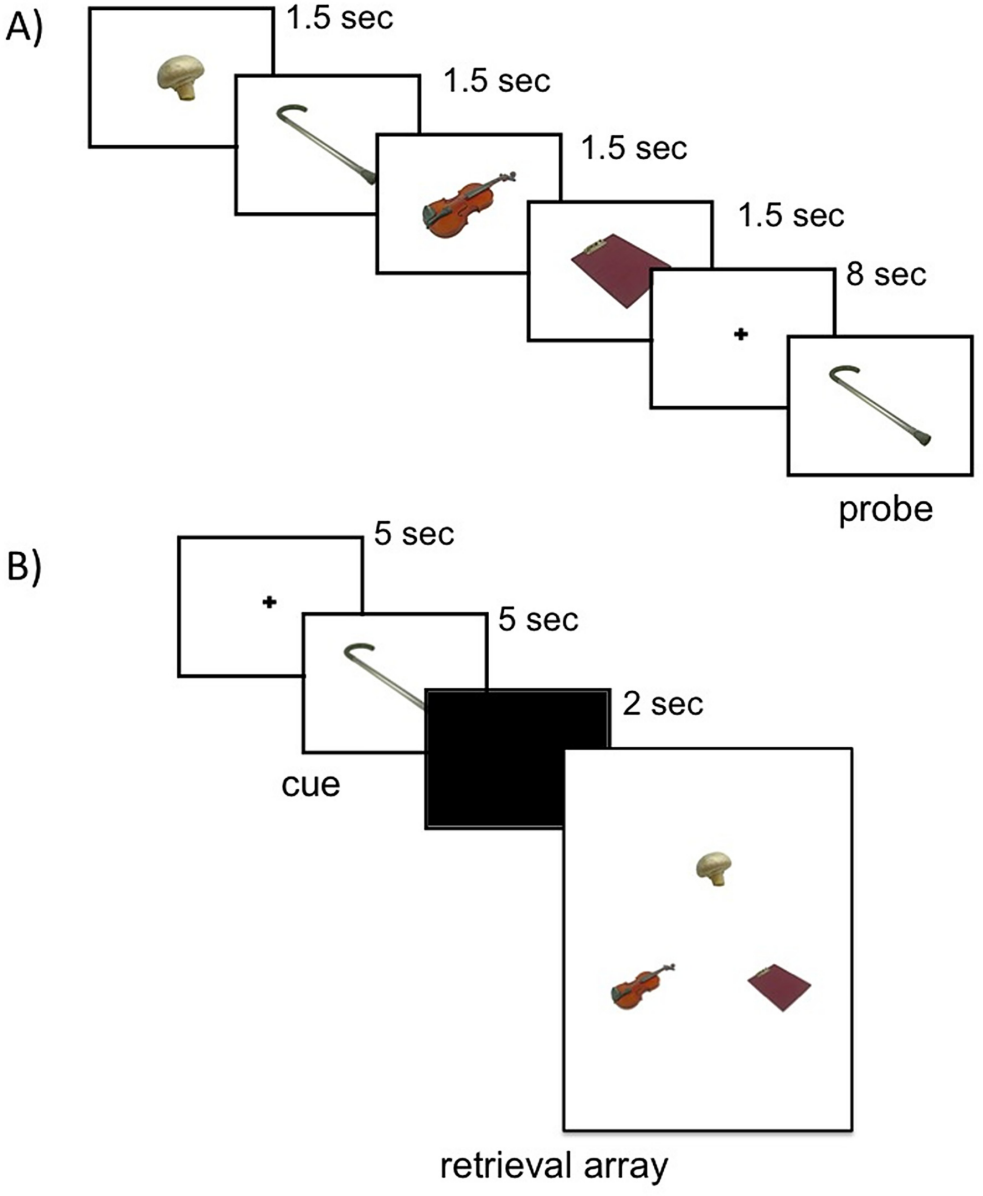
Sample temporal order trial in the encoding phase (A) and retrieval phase (B). At encoding, participants stated the ordinal position of the probe. At retrieval, participants selected the item from the retrieval array that they believe came immediately after the cue during the encoding phase.

From the stimulus set, we randomly selected 75 groups of 4 items (quadruplets) to be presented as encoding trials (Fig 1A). Within a quadruplet sequence, each object was presented alone in the center of the screen for 1.5 seconds. Participants were asked to remember the order of items presented within each quadruplet. Following the quadruplet sequence, participants were shown one of the objects from that quadruplet (probe item) and asked to select (via button press) whether it was the first, second, third or fourth object in the quadruplet sequence. The probe item remained on screen until response. We randomly selected which items from the sequence would be used as probes. Encoding trials were randomly split into three runs of 25 trials each, separated by a 1–2 minute break. Following encoding, participants were given a 10-minute break.

#### Retrieval phase

Participants were told that they would see one of the objects from the encoding phase (cue object; same object as probe item from encoding phase), and were asked to choose the object that came immediately after that object in the sequence (target object) from an array of three objects (Fig 1B). From the 75 probe items from the encoding phase, 60 had been presented either in the first, second or third position in the quadruplet sequence and could be used to probe memory for the item that came after it. Fifteen probe items from the encoding phase had been presented in the fourth position and thus could not be used in the retrieval phase (since no object came immediately after it within a sequence); we included these trials to ensure that participants attended to the whole sequence of 4 items during encoding. For each retrieval trial array, one object was the target (object that followed the retrieval cue), and the other two objects were distracters. The spatial placement (left, right, top) of the target object in the retrieval array was randomly selected with the constraint that the target object would be in each of the three positions an equal amount of times across trials. The retrieval array remained on screen until response.

The nature of the distracters in the retrieval array changed as a function of experimental condition. Although participants were asked to establish temporal order across conditions, differences in distracter type affected how order temporal could be retrieved. In the *temporal order* condition, the distracters were from the same sequence as the cue and target; therefore, participants could only establish what object followed the cue object by recollecting the precise temporal order with which objects within a quadruplet were presented. In the *temporal context* condition, the distracters were selected from other sequences previously shown during the encoding task; therefore, participants could establish what object followed the cue by recollecting which object was presented around the same time as the cue object. In the *mixed condition*, one distracter was selected from the same sequence as the cue and target object; the other distracter was randomly selected from another sequence; therefore, both precise order and broader contextual information could be used to establish temporal order.

Overall the temporal order condition contained the maximum amount of information from encoding context (all items in retrieval array were presented during encoding of trial). The temporal context condition contained minimum reinstatement of encoding context (retrieval array contained only 1 item presented at encoding for trial). The mixed condition contained a medium amount of information from encoding context (retrieval array contained 2 items presented at encoding for trial). If the degree of contextual reinstatement influences performance in this paradigm then we should see different levels of overt accuracy, and differences in eye-movement patterns across the temporal order, mixed and temporal context conditions.

The 60 retrieval trials were divided equally across conditions. Trial orders were randomized and split into three runs (20 trials each), separated by a 1–2 minute break. The randomization procedures described throughout this section were performed twice to create two randomized stimulus sets of encoding and retrieval phase versions that cycled across participants. To ensure participants understood each task, participants were given practice encoding trials immediately before the encoding phase, and practice retrieval trials immediately before the retrieval phase. Note that the size of the objects varied between encoding phase and retrieval phase. Specifically, each individual object in the retrieval array was presented larger in the encoding phase. Thus it is unlikely that any eye-movement effects found are driven solely by a perceptual match between encoding and retrieval. The visual angle of each retrieval array image was 23.5 x 17.8 degrees. The visual angle of each of the three areas of interest (AOI) within the retrieval array was 7.1 x 8.1 degrees; each of the three objects were centered in its respective AOI and AOIs did not overlap.

## Results

### Overt Response Accuracy

#### Encoding phase

Accuracy was very high during the working memory task, M = 96.14, SD = 3.50, and did not differ as a function of experimental condition, *F*(2, 72) = 1.09, *p* = .34. Nevertheless, only correct trials from the encoding phase were included in analyses in the retrieval phase.

#### Retrieval phase

**Fig 2:**
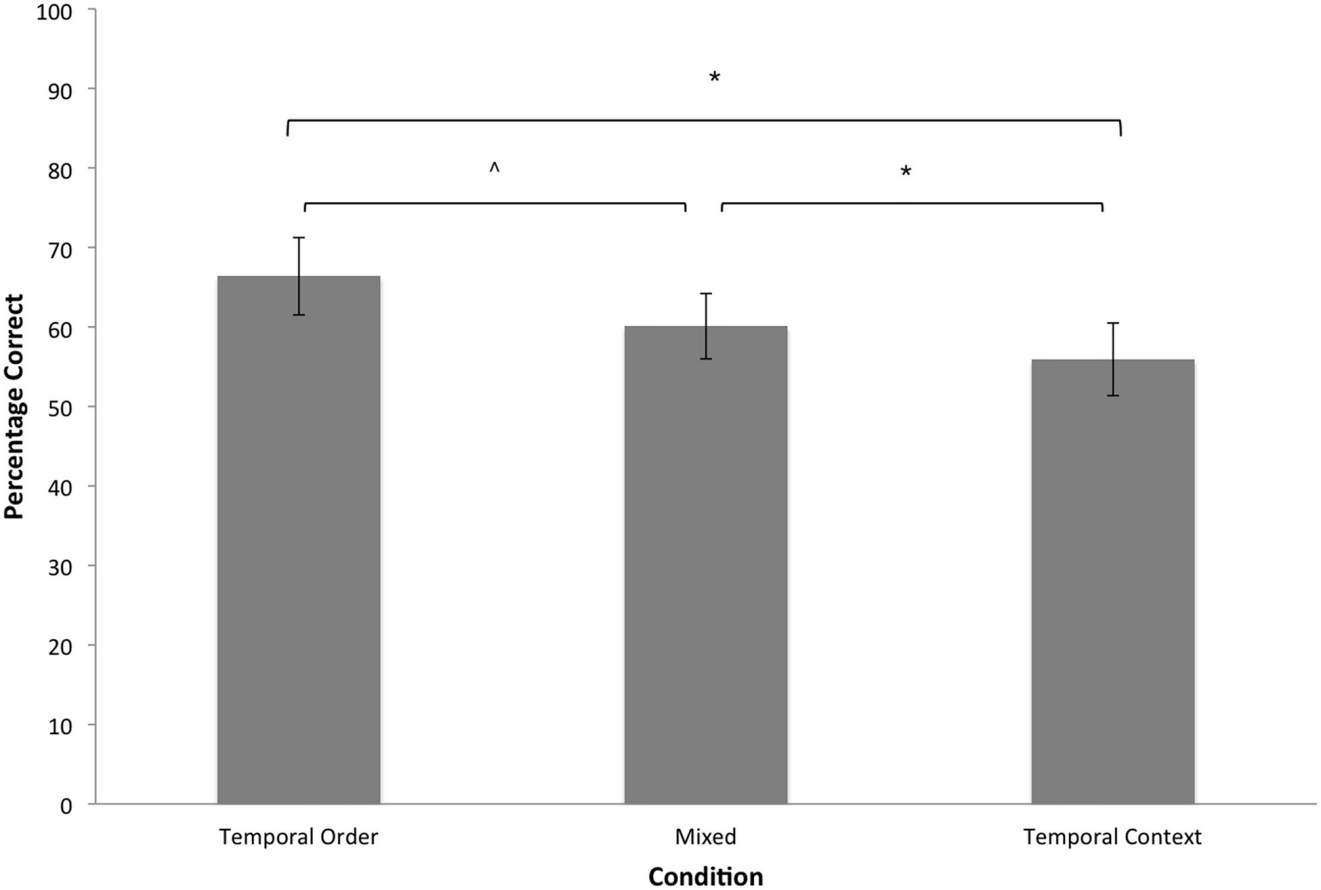
Experiment 1: Mean accuracy of overt decisions for each condition. Error bars represent 95% confidence intervals. * *p* < .05, ^ *p* ≤ .10.

A repeated measures ANOVA revealed a main effect of Condition, *F*(2, 72) = 9.94, *p* < .0001, *η*_p_^2^ = .22. As shown in Fig 2, accuracy in the temporal order condition was higher than accuracy in the mixed condition, which in turn was higher than accuracy in the temporal context condition. Thus, we replicated previous findings [40] in which accuracy in the temporal order condition was higher than accuracy in the temporal context condition. Additionally, the results of the mixed condition suggest there is a boost in performance when there is an increase in contextual cues that could aid reinstatement of temporal order.

### Temporal Memory Response Times

All participants took several seconds to respond to each trial (see Table 1). An Accuracy (correct, incorrect) x Condition (temporal order, temporal context) ANOVA revealed a main effect of Accuracy, *F*(1, 36) = 31.13, *p* < .0001, *η*_p_^2^ = .46. Response times were faster for correct than incorrect trials. There was no main effect of Condition, *F*(2, 72) = .69, *p* = .50, *η*_p_^2^ = .02, or interaction, *F* = 1.2, *p* = .30, *η*_p_^2^ = .03. This task is demanding and decisions take several seconds.

**Table 1:** Response times (in milliseconds) for each condition in Experiment 1.

Condition	Correct Trials Mean (SD)	Incorrect Trials Mean (SD)
Temporal Order	5150 (1910)	6547 (3219)
Mixed	5484 (2470)	6382 (2439)
Temporal Context	5572 (2301)	6440 (2697)

### Temporal Memory Eye Movements

Consistent with previous research [3, 40], we examined whether participants spent more time viewing selected objects in correct trials compared to selected objects in incorrect trials. Disproportionate viewing for correct trials compared to incorrect trials would suggest that eye movements reflected veridical memory. We calculated the proportion of viewing to the selected object for each time bin by summing the duration of fixations made to the selected object and dividing that by the sum of durations made to all AOIs (target + distracter 1 + distracter 2). By definition, the selected object was the target for correct trials, and was a distracter for incorrect trials.

#### Full trial length

An Accuracy x Condition repeated-measures ANOVA was conducted to determine if there was disproportionate viewing to the target object compared to the incorrectly selected distracter across the full trial length, which was variable across participants depending on when they responded. This analysis revealed a main effect of Accuracy, *F*(1, 36) = 19.31, *p* < .001, no main effect of Condition (*p* = .37), and an Accuracy x Condition interaction, *F*(2, 72) = 3.05, *p* = .05, *η*_p_^2^ = .08. Follow-up analysis found that there was disproportionate viewing to the selected item for correct trials compared to incorrect trials in the temporal order condition, *t*(36) = 3.99, *p* < .0005, but not in the temporal context condition, *t*(36) = 1.22, *p* = .23, replicating the results of Pathman and Ghetti [40]. In the mixed condition, disproportionate viewing for correct trials approached statistical significance, *t*(36) = 1.93, *p* = .06. Next we examined the time course of early eye movements. The examination of timing of these effects is important because it can inform us about how early these eye-movement effects were visible.

#### Time course of early eye movements

**Fig 3:**
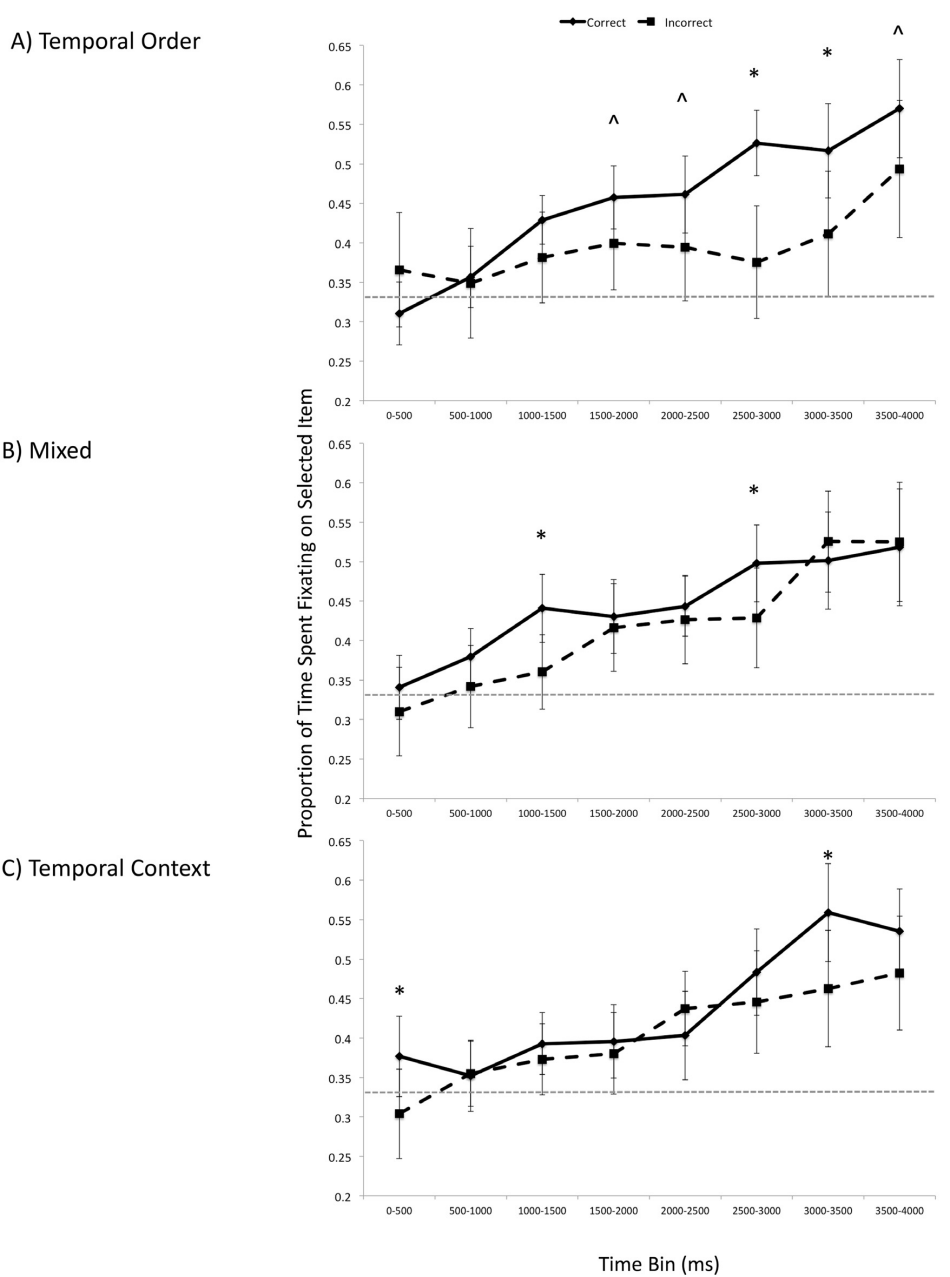
Experiment 1: Time course of looking in the early phase of the retrieval trial array for the temporal order condition (A), mixed condition (B), and temporal context condition (C). Error bars represent 95% confidence intervals. * *p* < .05, ^ *p* ≤ .10.

Paralleling Pathman and Ghetti [40], we examined fixations up to 4000 ms after stimulus onset, in 500 ms bins. This maximum time point was selected such that it was later than those reported in previous eye movement investigations of memory for context in adults [2–3], but earlier than when participants made their button-press responses. An Accuracy (correct, incorrect) x Condition x Time Bin ANOVA revealed a main effect of Accuracy, *F*(1, 34) = 18.99, *p* < .001, *η*_p_^2^ = .36, and a main effect of Time Bin, *F*(7, 238) = 23.50, *p* < .0001, *η*_p_^2^ = .41, and a Accuracy x Condition x Time Bin interaction, *F*(14, 476) = 1.91, *p* < .05, *η*_p_^2^ = .05. As shown in Fig 3, eye movements reflected veridical memory for temporal information for multiple time bins in the temporal order condition (1500–2000 ms: *t*(36) = 1.66, *p* < .10; 2000–2500 ms: *t*(36) = 1.96, *p* < .06; 2500–3000 ms: *t*(36) = 3.92, *p* < .0005; 3000–3500 ms: *t*(36) = 2.43, *p* < .05; 3500–4000 ms: *t*(36) = 1.84, *p* < .10). In the mixed condition, there were two time bins in which there was disproportionate viewing for correct trials compared to incorrect trials (1000–1500 ms: *t*(36) = 2.60, *p* < .05; 2500–3000 ms: *t*(36) = 2.20, *p* < .05). In the temporal context condition, one late temporal bin showed the eye-movement effect (3000–3500 ms: *t*(36) = 2.62, *p* < .05). Note that although there was a difference between correct and incorrect trials in the first time bin (0–500 ms: *t*(36) = 2.17, *p* < .05), each of these values hovers around chance (as shown in Fig 3) and thus cannot be clearly interpreted.

Overall, across the first 4-seconds since the onset of the test trial, there was greater looking at selected items for correct trials compared to selected but incorrect trials for the temporal order and mixed conditions (temporal order: *t*(36) = 4.35, *p* < .0005, Cohen’s *d* = .71; mixed: *t*(36) = 3.61, *p* = .005, Cohen’s *d* = .59; temporal context *t*(36) = 1.97, *p* = .06, Cohen’s *d* = .32). This was not the case in the second 4-second interval for all conditions (temporal order: *t*(36) = .65, *p* = .52; mixed: *t*(36) = -.81, *p* = .42; temporal context *t*(36) = .62, *p* = .54), suggesting that in the later part of the trials eye gazes no longer reflect accuracy, but response selection. See S1 Fig.

In sum, eye movements reflected veridical memory for temporal order, but not temporal context, seconds before this selection was made. The eye-movement effects and accuracy levels of the mixed condition were in between those of the temporal order and temporal context condition, suggesting that reinstatement of the original temporal sequence may account in part for the results.

These results suggest that temporal order may be a more dominant organizational principle than temporal context, but raise an additional question about the nature of this organization. The three conditions in this experiment not only differed in the extent to which the distractors in the retrieval array reinstated the encoding phase quadruplet, but also in the extent to which they included items representing a unique ordinal position from their respective encoding phase quadruplet. In other words, in the temporal order condition, all of the retrieval array items included items that were in their own unique ordinal position in the studied sequences (because, being all selected from one studied quadruplet, they necessarily held distinct ordinal positions); in the mixed condition this was the case of two of the retrieval array items (i.e., the target and one of the distracters,); in the temporal context condition the distractors were not selected to have held distinct ordinal positions in their original sequence. This is important in light of recent evidence showing that the hippocampus is involved in retrieving the bound representation of an item and its ordinal position across different sequences [45], suggesting a mechanism through which the hippocampus encodes for absolute ordinal position (in the present study sequence: 1^st^, 2^nd^, 3^rd^, 4^th^). Experiment 2 was conducted to assess whether absolute ordinal position could account for the results of this experiment.

## Experiment 2

The goal of Experiment 2 was to test the hypothesis that memory for ordinal position of items from the encoding phase could explain differences in eye-movements effects and increased accuracy in the temporal order compared to temporal context condition in Experiment 1. Here we included temporal order, temporal context and mixed conditions, like Experiment 1. However, we put a constraint on how distracters were selected for the temporal context and mixed conditions: all items were selected such that they were from a unique ordinal position in their respective encoding sequences. Thus, if eye-movement effects and increased accuracy in the temporal order condition depended on differences in the absolute ordinal position represented within each test trial, then we would expect that differences among conditions would be eliminated when all items had unique ordinal positions across experimental conditions. On the other hand, if temporal order, in the absence of item-ordinal position binding, was the most critical factor, the results of Experiment 2 should fully replicate those of Experiment 1.

### Participants

39 young adults (*M* = 20.93 years, *SD* = 2.77; 51.3% females) took part in the study. No participants were excluded because of chance performance in the retrieval phase. Like Experiment 1, this was a convenience sample, with sample size comparable with previous eye movement investigations [2, 36].

### Procedures

Methods were identical to those of Experiment 1. The only exception was that distracters were selected such that they had a unique ordinal position. The *temporal order condition* was identical to all previous experiment; by definition, all objects had a unique ordinal position. In the *temporal context condition*, the two distracters were randomly selected from other sequences, like in the previous experiment; however there was a constraint such that each selected distracter must have a unique ordinal position when put together with the target and cue. For example, if the cue and target were in ordinal positions 2 and 3 respectively in the encoding phase, then one distracter was selected from another sequence from ordinal position 1, and the other sequence was selected from another sequence from ordinal position 4. This same constraint was imposed for the distracter from another sequence in the *mixed condition*.

## Results

### Overt Response Accuracy

#### Encoding phase

As in Experiment 1, accuracy was very high in working memory task, M = 94.46, SD = 5.39, and did not differ as a function of retrieval condition, *F*(2, 76) = 1.88, *p* = .16. Nevertheless, only correct trials from the encoding phase were included in analyses in the retrieval phase.

#### Retrieval phase

**Fig 4:**
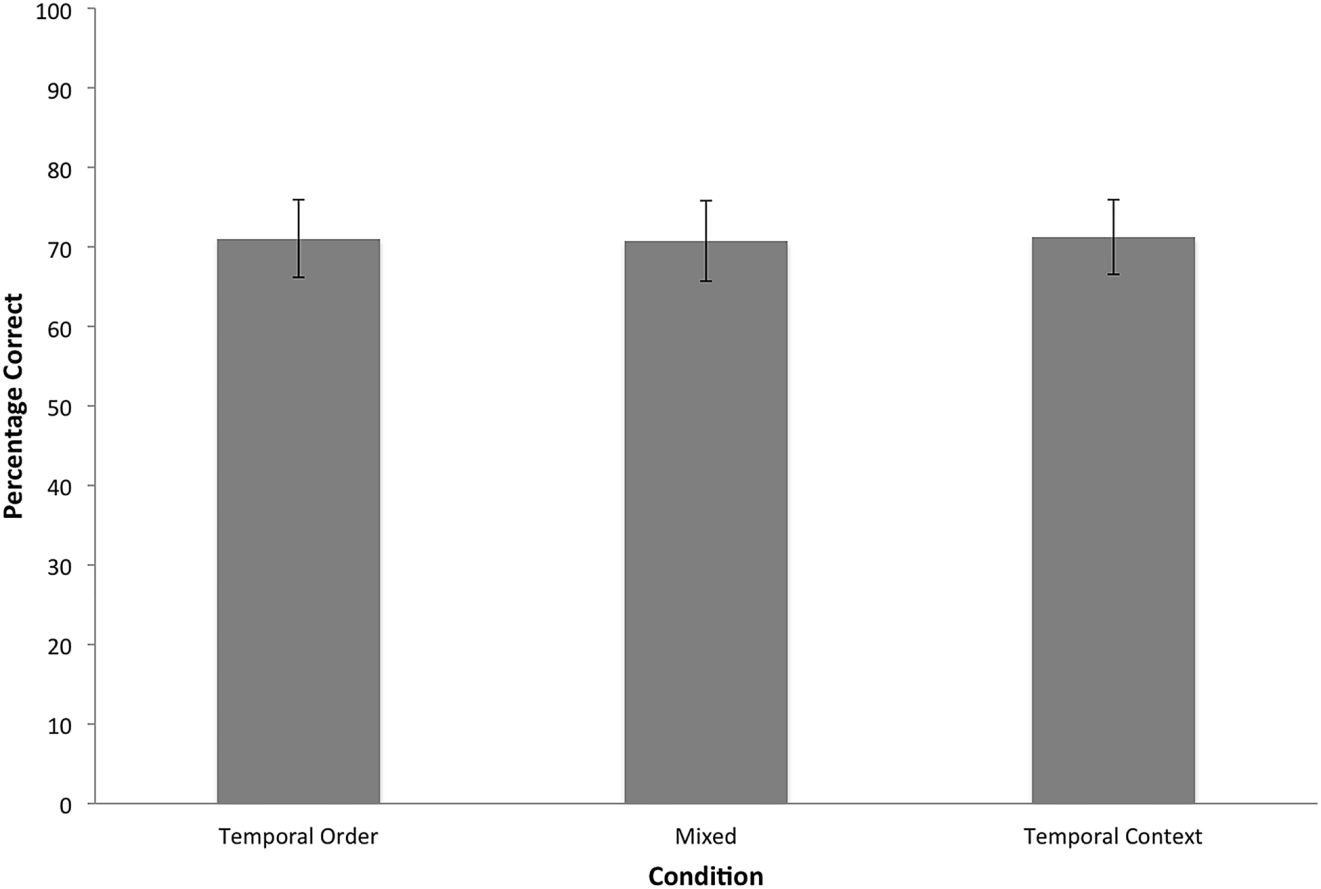
Experiment 2: Mean accuracy of overt decisions for each condition. Error bars represent 95% confidence intervals. * *p* < .05.

A repeated measures ANOVA revealed no main effect of Condition, *F*(2, 76) = .02, *p* = .98. As shown in Fig 4, accuracy did not differ across conditions. These results are consistent with the hypothesis that retrieval of an item’s ordinal position at encoding accounted for greater accuracy in the temporal order condition compared to the temporal context condition in Pathman and Ghetti [40] and Experiment 1.

### Temporal Memory Response Times

All participants took several seconds to respond to each trial (see Table 2). An Accuracy (correct, incorrect) x Condition (temporal order, temporal context) ANOVA revealed a main effect of Accuracy, *F*(1,35) = 18.42 *p* < .001, *η*_p_^2^ = .35. Response times were faster for correct than incorrect trials. Response times did not differ across conditions overall, *F*(2, 70) = 1.27, *p* = .29, *η*_p_^2^ = .03, however there was an accuracy x condition interaction *F*(2, 70) = 3.69, *p* < .05, *η*_p_^2^ = .10. Follow-up showed that there were no response time differences across conditions for correct trials, *F*(2, 76) = 2.05, *p* = .14, but a response time difference across conditions approached significance for incorrect trials, *F*(2, 70) = 2.91, *p* = .06. *η*_p_^2^ = .08. (i.e., response times in the mixed condition were longer compared to the temporal order condition for incorrect trials.)

**Table 2:** Response times (in milliseconds) for each condition in Experiment 2.

Condition	Correct Trials Mean (SD)	Incorrect Trials Mean (SD)
Temporal Order	5432 (2681)	6064 (2381)
Mixed	5234 (3008)	6777 (3282)
Temporal Context	5519 (2413)	6333 (2457)

### Temporal Memory Eye Movements

#### Full trial length

An Accuracy x Condition ANOVA of proportion of time spent viewing the selected item revealed a main effect of Accuracy, *F*(1, 35) = 29.74, *p* < .001, no effect of Condition (*p* = .33), and no Accuracy x Condition interaction (*p* = .61). Across conditions, there was disproportionate viewing to the selected distracter for correct compared to incorrect trials.

Like in Experiment 1, and previous studies [2,3, 11–13] we next examined the time course of effects soon after stimulus onset.

#### Time course of early eye movements

**Fig 5:**
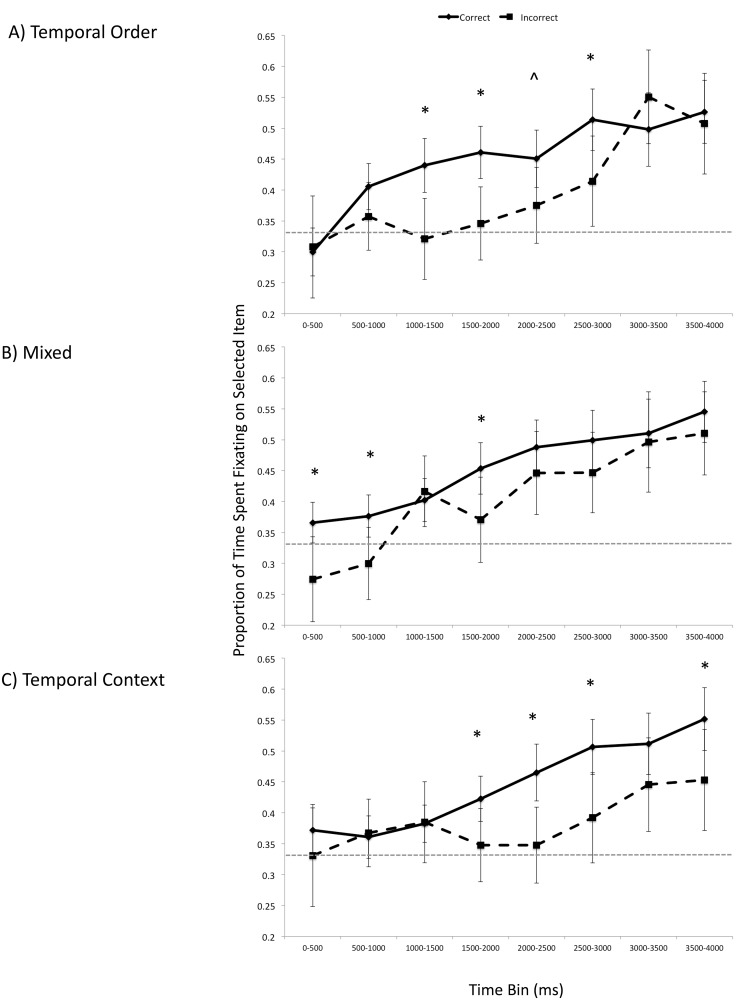
Experiment 2: Time course of looking in the early phase of the retrieval trial array for the temporal order condition (A), mixed condition (B), and temporal context condition (C). Error bars represent 95% confidence intervals. * *p* < .05, ^ *p* ≤ .10.

An Accuracy (correct, incorrect) x Condition x Time Bin ANOVA revealed only a main effect of Accuracy, *F*(1, 30) = 25.53, *p* < .0001, *η*_p_^2^ = .47, and a main effect of Time Bin, *F*(7, 210) = 29.21, *p* < .0001, *η*_p_^2^ = .49. As shown in Fig 5, eye movements showed disproportionate viewing to the selected item for correct trials within the first 2 seconds for all conditions. It should be noted that the eye-movement effect occurred earlier for the temporal order condition, compared to the temporal context condition. For the temporal order condition eye-movement effects were found in the 1000–1500 ms bin, *t*(37) = 2.74, *p* < .01, 1500–2000 ms bin, *t*(37) = 3.03, *p* < .005, 2000–2500 ms bin, *t*(37) = 1.77, *p* < .10, and the 2500–3000 ms bin, *t*(37) = 2.54, *p* < .05. In the mixed condition, an eye-movement effect was found in the 1500–2000 ms time bin, *t*(35) = 2.21, *p* < .05. For the temporal context condition disproportionate viewing for correct compared to incorrect trials were found in the 1500–2000 ms bin, *t*(36) = 2.28, *p* < .05, 2000–2500 ms bin, *t*(36) = 3.72, *p* < .001, 2500–3000 ms bin, *t*(36) = 3.03, *p* < .005, and the 3500–4000 ms bin, *t*(35) = 2.30, *p* < .05.

As in Experiment 1, greater looking at selected items for correct trials compared to selected incorrect trials was evident in the first 4-second interval for all conditions (temporal order: *t*(36) = 4.10, *p* < .0005, Cohen’s *d* = .66; mixed: *t*(36) = 4.82, *p* < .0005, Cohen’s *d* = .80; temporal context *t*(36) = 4.25, *p* < .0005, Cohen’s *d* = .70), but not in the second 4-second interval for all conditions (temporal order: *t*(36) = 1.38, *p* = .18; mixed: *t*(36) = .004, *p* = 1.0; temporal context: *t*(36) = 1.26, *p* = .21). Again, eye-movement effects reflecting accuracy (not selection) are restricted to the early part of the trial. See S2 Fig.

The results of Experiment 2 are consistent with the hypothesis that eye movement effects reflect binding of an item to absolute ordinal position; memory for ordinal position was responsible for the more apparent eye-movement effects in the temporal order condition compared to the temporal context condition in Experiment 1.

## Discussion

The present studies aimed to examine whether eye movements can capture temporal features of memory episodes. Across two experiments we identified specific eye-movement signatures that reflected successful retrieval of memory for temporal order in long-term memory. Adults showed eye-movement effects associated with veridical memory for temporal order as early as 1000 to 1500 ms after stimulus onset, several seconds before participants made overt order judgments. The current research establishes that eye movements are useful to examine one of the defining features of episodic memory.

Importantly, we determined what aspects of temporal order processing were reflected in eye movements. In Experiment 1, we found that eye-movement effects were restricted to the temporal order condition. Experiment 1 showed that increasing the contextual cues (mixed condition) resulted in the emergence of some eye-movement effects and an increase in memory accuracy. This finding extends to eye movements the finding that temporal contiguity of cues aides temporal order recall [46].

Experiment 2, however, showed that memory for absolute ordinal position was more critical: manipulating the retrieval array items such that each item was from a unique ordinal position brought about eye-movement effects in all conditions and eliminated accuracy differences among them. These results suggest that the eye-movement effects are based on binding an event to its absolute ordinal position within a quadruplet, suggesting that ordinal position served as an organizational principle that occurred across all temporal memory conditions. This finding highlights time-tagging theories, which posit that during encoding events are marked with “tags” of time or ordinal position ([14, 47]; for review see [48]). It is also consistent with recent studies in which neural activity is associated with object-ordinal position binding during memory retrieval [45].

In this paradigm, the retrieval of temporal information guided decision making (which of the three items to choose), and both of these sets of processes were reflected in eye movements. We cannot precisely separate initial reinstatement or retrieval of temporal information from decision processes using eye movement measures. Still, we can determine how eye movements change from stimulus onset to overt response, and see the progression of pattern differences. Analysis of the early portion of the trial compared to the later portion of the trial showed that, early eye movements predicted accuracy in overt choice, but later eye movements did not (participants spent more time looking at the selected items, irrespective of accuracy). In addition, we note that eye movements provided information that did not always coincide with response times. For example, Experiment 1, eye movement effects were reported in the temporal order condition but not in other conditions, and yet response times did not differ across the three conditions, nor was there an interaction between condition and accuracy. Thus it is not simply that the presence or absence of eye movement effects is based on shorter or longer response times or decision-making time. Thus, eye movements provided unique information that can be used to complement measures of response times and accuracy to determine the mechanisms supporting memory retrieval. By the same token, Saint-Aubin, Tremblay and Jalbert [49] argue that eye movement measures complement recall measures, providing non-redundant information, in short-term memory tasks.

Future research should examine how task instructions affected our results because this has implications for the automaticity of temporal order encoding [50]. Previous work (in which there was no delay between study and test) has shown that ordinal position of items within lists are remembered and do not depend on intentional processing of temporal information [51–52]. In the present research, although participants did not know they would be tested on temporal memory after a long-term delay, ordinal position was explicitly attended during learning. We do not know whether ordinal position would have been encoded and retained if our task instructions were not related to temporal order. If encoding task instructions do not need to be about temporal order, this would provide further evidence for time-tagging theories that posit that temporal information during encoding is laid down automatically [47]; currently relatively little is known about the nature of automatic temporal tags (see [16, 48] for discussion), but a role of these tags would challenge currently held views that reconstruction processes (using other contextual information, such as environmental cues, to infer when an event occurred) [16], not time-tagging, are the predominant way in which human adults remember “when” information [16, 48].

The present investigation adds to the corpus of studies that have used eye movements to investigate aspects of temporal memory over short delays. Unlike the present investigation, these other studies involve visual-spatial displays during encoding, and thus were not intended to be “pure” measures of temporal order. Ryan and Villate [53] presented participants with three objects presented one at a time, each in a different location on a computer screen. After a 2-second delay, all three objects were presented simultaneously and researchers found evidence that participants inspected the objects in the order with which they had been originally presented. Moreover, Tremblay, Saint-Aubin and Jalbert [54] found that recall accuracy was higher when participants “replayed” the temporal order of items via eye movements during the delay between study and test (but see Godijn & Theeuwes [55] for contradictory findings). Together, these findings show that eye-movement patterns during delay [31, 54] and test [53] may support short-term memory for temporal order. This work, when combined with results of the present investigation, suggests that memory-guided eye movements can help elucidate different aspects of short-term memory, or long-term episodic memory [10], like binding of events to time and space. Future work could examine whether “replay” via eye movements is related to sequential neuronal firing patterns that have been found to be associated with temporal memory across delays in animal models [56]. Further work could also examine how short-term memory effects in which temporal order is “replayed” via eye movements is compared to the eye-movement effects reported in the present investigation. For example, a future study could modify the present paradigm such that during the encoding phase, each object is presented at a different location on the screen, and then fixations could be examined during the short-term delay. Any short-term memory eye movement effects could be tested for relations with either overt long-term recall or eye-movement effects (e.g., amount of difference between selected correct and incorrect looking fixation durations, or, onset of the eye- movement effects). To our knowledge, no study has examined whether rehearsal of ordered presentation of items via eye movements (whether overt or covert) is related to long-term retention of temporal order of the items via eye movements or explicit recall. Such a study would support claims that eye movements are a mechanism by which information is “bound into a lasting representation, and by which current information is compared to stored representations” ([53], p. 267).

In summary, across two experiments, we established that eye movements can reflect veridical temporal memory over a long-term delay. These findings adds to the promise of eye movements being used to assess, and directly compare, memory across populations including nonverbal infants, typical children and adults, patients with psychiatric or neurodegenerative disorders, and nonhuman primates. Moreover, although episodic memory is traditionally considered a type of memory that is expressed deliberately and is associated with subjective experience of remembering [15], these findings, along with other investigations of eye movements [1–3] suggest that some part of the memory representation may be accessed before it is readily available for overt decisions and reports, which motivate new questions about how to conceptualize episodic memory.

## Supporting Information

S1 Fig Experiment 1 Eye Movement Effects from 0–4 Seconds and 4–8 Seconds. Error bars represent +/- standard error. * *p* < .05, ^ *p* < .07.
(TIF)

S2 Fig Experiment 2 Eye Movement Effects from 0–4 Seconds and 4–8 Seconds. Error bars represent +/- standard error. * *p* < .05.
(TIF)

## References

[1] Hannula DE, Baym CL, Warren DE, Cohen NJ. The eyes know: Eye movements as a veridical index of memory. Psychological Science 2012; 23:278–287. 10.1177/0956797611429799 22327015 PMC3917553

[2] Hannula DE, Ryan JD, Tranel D, Cohen NJ. Rapid onset relational memory effects are evident in eye movement behavior, but not in hippocampal amnesia. Journal of Cognitive Neuroscience 2007; 19:1690–1705. 17854282 10.1162/jocn.2007.19.10.1690

[3] Hannula DE, Ranganath C. The eyes have it: hippocampal activity predicts expression of memory in eye movements. Neuron 2009; 63:592–599. 10.1016/j.neuron.2009.08.025 19755103 PMC2747814

[4] Beck MR, Peterson MS, Angelone BL. The roles of encoding, retrieval, and awareness in change detection. Memory & Cognition 2007; 35:610–620. 17848019 10.3758/bf03193299

[5] Henderson JM, Hollingsworth A. Eye movements and visual memory: Detecting changes to saccade targets in scenes. Perception & Psychophysics 2003; 65: 58–71. 12699309 10.3758/bf03194783

[6] Hollingsworth A, Williams CC, Henderson JM. To see and remember: Visually specific information is retained in memory from previously attended objects in natural scenes. Psychonomic Bulletin & Review 2001; 8:761–768. 11848597 10.3758/bf03196215

[7] Ryan JD, Cohen NJ. Processing and short-term retention of relational information in amnesia. Neuropsychologia 2004; 42:497–511. 14728922 10.1016/j.neuropsychologia.2003.08.011

[8] Ryan JD, Cohen NJ. The nature of change detection and on-line representations of scenes. Journal of Experimental Psychology: Human Perception and Performance 2004; 30:988–1015. 15462635 10.1037/0096-1523.30.5.988

[9] Ryan JD, Althoff RR, Whitlow S, Cohen NJ. Amnesia is a deficit in relational memory. Psychological Science 2000; 11:454–461. 11202489 10.1111/1467-9280.00288

[10] Hannula DE, Althoff RR, Warren DE, Riggs L, Cohen NJ, Ryan JD. Worth a glance: using eye movements to investigate the cognitive neuroscience of memory. Frontiers in Human Neuroscience 2010; 4:1–16. 10.3389/neuro.09.001.2010 21151363 PMC2995997

[11] Koski J, Olson IR, Newcombe NS. Tracking the eyes to see what children remember. Memory 2013; 21:396–407. 10.1080/09658211.2012.735241 23163586 PMC4783158

[12] Richmond J, Nelson CA. Relational memory during infancy: Evidence from eye tracking. Developmental Science 2009; 12:549–556. 10.1111/j.1467-7687.2009.00795.x 19635082

[13] Richmond JL, Power J. Age-related differences in memory expression during infancy: Using eye-tracking to measure relational memory in 6- and 12-month-olds. Developmental Psychobiology 2014; 56:1341–1351. 10.1002/dev.21213 24634167

[14] Tulving E. Episodic and semantic memory In: Tulving E, Donaldson W, editors. Organization of memory. New York: Academic Press; 1972 pp. 382–403.

[15] Tulving E. Episodic memory: from mind to brain. Annual Review Psychology 2002; 53:1–25. 11752477 10.1146/annurev.psych.53.100901.135114

[16] Friedman WJ. Memory for the time of past events. Psychological Bulletin 1993; 113: 44–66.

[17] Marshuetz C. Order information in working memory: an integrative review of evidence from brain and behavior. Psychological Bulletin 2005; 131:323–339. 15869331 10.1037/0033-2909.131.3.323

[18] Liversedge S, Gilchrist I, Everling S. Oxford Handbook of Eye Movements. New York: Oxford University Press; 2011.

[19] Rayner K. Eye movements and attention in reading, scene perception, and visual search. The Quarterly Journal of Experimental Psychology 2009; 62:1457–1506. 10.1080/17470210902816461 19449261

[20] Van der Stigchel S, Meeter M, Theeuwes J. Eye movement trajectories and what they tell us. Neurosci. Biobehav. Rev. 2006; 30:666–679. 16497377 10.1016/j.neubiorev.2005.12.001

[21] Kukona A, Altmann G, Kamide Y. Knowing what, where, and when: Event comprehension in language processing. Cognition 2014; 133: 25–31. 10.1016/j.cognition.2014.05.011 24955885

[22] Hooge ITC, Erkelens CJ. Control of fixation during a simple search task. Perception and Psychophysics 1996; 58:969–976. 8920834 10.3758/bf03206825

[23] Hoover MA, Richardson DC. When facts go down the rabbit hole: Contrasting features and objecthood as indexes to memory. Cognition 2008; 108:533–542. 10.1016/j.cognition.2008.02.011 18423431

[24] Theeuwes J, Belopolsky A, Olivers CNL. Interactions between working memory, attention and eye movements. Acta Psychologica 2009; 132:106–114. 10.1016/j.actpsy.2009.01.005 19233340

[25] Carlson-Radvansky LA. Memory for relational information across eye movements. Perception & Psychophysics 1999; 61:919–934. 10499004 10.3758/bf03206906

[26] Foulsham T, Kingstone A. Fixation-dependent memory for natural scenes: An experimental test of scanpath theory. Journal of Experimental Psychology: General 2013; 142:41–56. 22506754 10.1037/a0028227

[27] Holm L, Mäntylä T. Memory for scenes: Refixations reflect retrieval. Memory & Cognition 2007; 35:1664–1674. 18062544 10.3758/bf03193500

[28] Irwin DE, Zelinsky GJ. Eye movements and scene perception: Memory for things observed. Perception & Psychophysics 2002; 64:882–895. 12269296 10.3758/bf03196793

[29] Johansson R, Johansson M. Look here, eye movements play a functional role in memory retrieval. Psychological Science 2014; 25:236–242. 10.1177/0956797613498260 24166856

[30] Laeng B, Bloem IM, D’Ascenzo S, Tommasi L. Scrutinizing visual images: the role of gaze in mental imagery and memory. Cognition 2014; 131: 263–283. 10.1016/j.cognition.2014.01.003 24561190

[31] Olsen RK, Chiew M, Buchsbaum BR, Ryan JD. The relationship between delay period eye movements and visuospatial memory. Journal of Vision 2014; 14:1–11. 10.1167/14.14.1 24403394

[32] Zelinsky GJ, Loschky LC. Eye movements serialize memory for objects in scenes. Perception & Psychophysics 2005; 67:676–690. 16134461 10.3758/bf03193524

[33] Hayhoe MM, Bensinger DG, Ballard DH. Task constraints in visual working memory. Vision Research 1997; 38:125–137. 9474383 10.1016/s0042-6989(97)00116-8

[34] Smith CN, Hopkins RO, Squire LR. Experience- dependent eye movements, awareness, and hippocampus-dependent memory. Journal of Neuroscience 2006: 26:11304–11312. 17079658 10.1523/JNEUROSCI.3071-06.2006 PMC2424210

[35] Schwedes C, Wentura D. The revealing glance: Eye gaze behavior to concealed information. Memory & Cognition 2012; 40:642–651. 22194248 10.3758/s13421-011-0173-1

[36] Ryan JD, Hannula DE, Cohen NJ. The obligatory effects of memory on eye movements. Memory 2007;15:508–525. 17613794 10.1080/09658210701391022

[37] Holm L, Eriksson J, Andersson L. Looking as if you know: Systematic object inspection precedes object recognition. Journal of Vision 2008; 8:1–7. 10.1167/8.4.14 18484853

[38] Kumaran D, Wagner AD. It’s in My Eyes, but it doesn’t look that way to me. Neuron 2009; 63:561–3. 10.1016/j.neuron.2009.08.027 19755098

[39] Fortin NJ, Agster KL, Eichenbaum HB. Critical role of the hippocampus in memory for sequences of events. Nature Neuroscience 2002; 5:458–462. 11976705 10.1038/nn834 PMC4053170

[40] Pathman T, Ghetti S. The eyes know time: A novel paradigm to examine the development of temporal memory. Child Development 2014; 85:792–807. 10.1111/cdev.12152 23962160

[41] Ezzyat Y, Davachi L. Similarity breeds proximity: Pattern similarity within and across contexts is related to later mnemonic judgments of temporal proximity. Neuron 2014; 81:1179–1189. 10.1016/j.neuron.2014.01.042 24607235 PMC3983791

[42] Tubridy S, Davachi L. Medial temporal lobe contributions to episodic sequence encoding. Cerebral Cortex 2011; 21:272–280. 10.1093/cercor/bhq092 20494967 PMC3020579

[43] Brodeur MB, Dionne-Dostie E, Montreuil T, Lepage M. The bank of standardized stimuli (BOSS), a new set of 480 normative photos of objects to be used as visual stimuli in cognitive research. PLOS ONE 2010; 5:e10773 10.1371/journal.pone.0010773 20532245 PMC2879426

[44] Tobii Technology. WhitePaper: Timing guide for Tobii eye trackers and eye tracking software. Danderyd, Sweden: Tobii Technology AB; 2010.

[45] Hsieh LT, Gruber MJ, Jenkins LJ, Ranganath C. Hippocampal activity patterns carry information about objects in temporal context. Neuron 2014; 81:1165–1178. 10.1016/j.neuron.2014.01.015 24607234 PMC3984944

[46] Solway A, Murdock BB, Kahana MJ. Positional and temporal clustering in serial order memory. Memory & Cognition 2012; 40:177–190. 22057363 10.3758/s13421-011-0142-8 PMC3282556

[47] Hasher L, Zacks RT. Automatic and effortful processes in memory. Journal of Experimental Psychology: General 1979; 108:356–388.

[48] Friedman WJ. Time in autobiographical memory. Social Cognition 2004; 22:591–605.

[49] Saint-Aubin J, Tremblay S, Jalbert A. Eye movements and serial memory for visual-spatial information. Experimental Psychology 2007; 54:264–272. 17953146 10.1027/1618-3169.54.4.264

[50] Naveh-Benjamin M. Coding of temporal order information: An automatic process? Journal of Experimental Psychology: Learning, Memory and Cognition 1990; 16:117–126. 10.1037//0278-7393.13.4.595 2959742

[51] Toglia MP, Kimble GA. Recall and use of serial position information. Journal of Experimental Psychology: Human Learning and Memory 1976; 2:431–445. 932652

[52] Zimmerman J, Underwood BJ. Ordinal position knowledge within and across lists as a function of instructions in free-recall learning. The Journal of General Psychology 1968; 79:301–307. 5725295 10.1080/00221309.1968.9710477

[53] Ryan JD, Villate C. Building visual representations: the binding of relative spatial relations across time. Visual Cognition 2009; 17:254–272.

[54] Tremblay S, Saint-Aubin J, Jalbert A. Rehearsal in serial memory for visual-spatial information: Evidence from eye movements. Psychonomic Bulletin & Review 2006; 13:452–457. 17048730 10.3758/bf03193869

[55] Godijn R, Theeuwes J. Overt is no better than covert when rehearsing visuo-spatial information in working memory. Memory & Cognition 2012; 40:52–61. 21769706 10.3758/s13421-011-0132-x PMC3246584

[56] MacDonald CJ, Lepage KQ, Eden UT, Eichenbaum H. Hippocampal ‘time cells’ bridge the gap in memory for discontiguous events. Neuron 2011; 71:737–749. 10.1016/j.neuron.2011.07.012 21867888 PMC3163062

