# Supplementary figures and images for: Eye Movements Provide an Index of Veridical Memory for Temporal Order
Source: PLoS One. 2015 May 20;10(5):e0125648. doi: 10.1371/journal.pone.0125648 (PMC4439145; doi:10.1371/journal.pone.0125648)

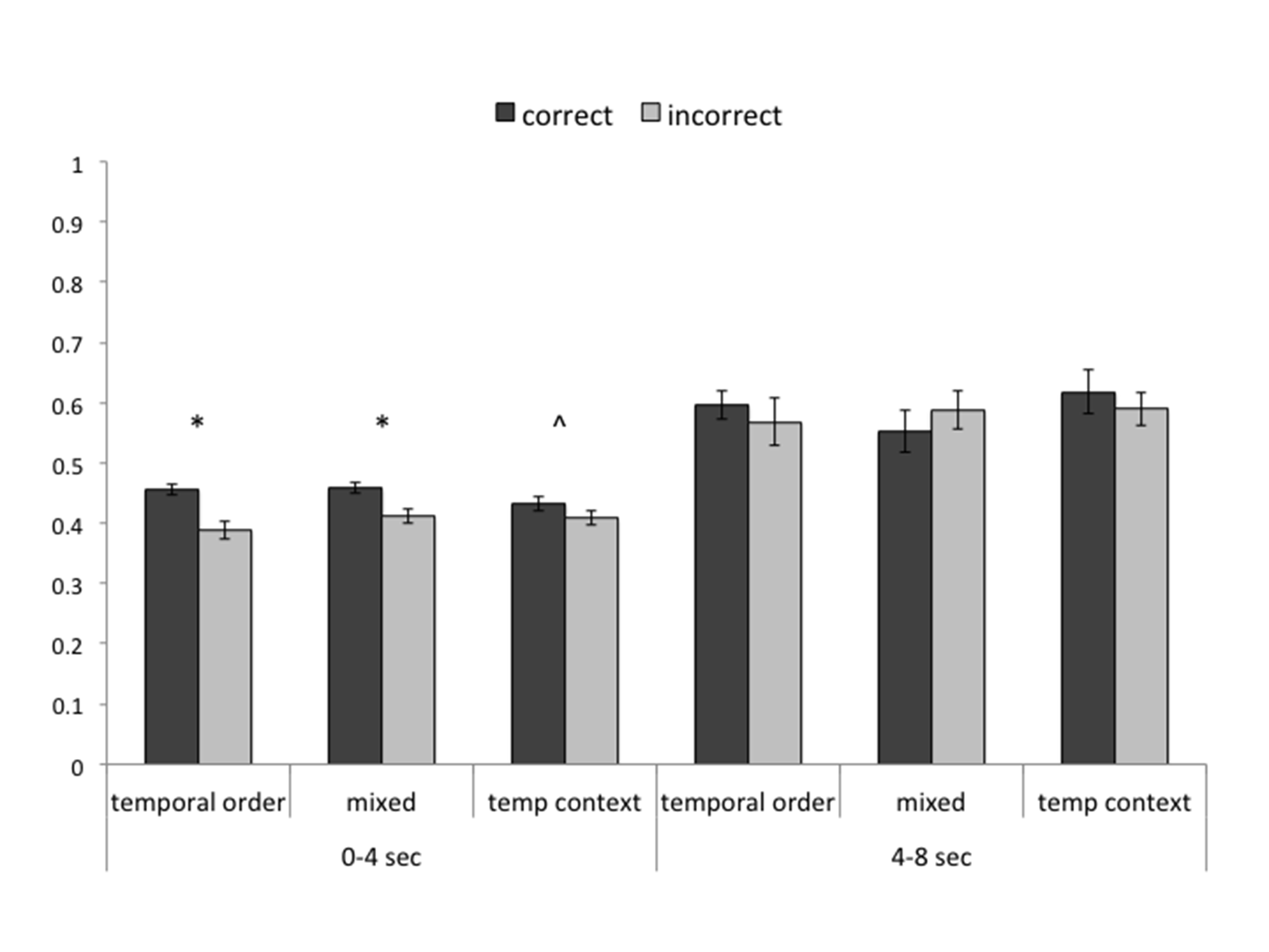

Supplement: S1 Fig — Error bars represent +/- standard error. * p < .05, ^ p < .07. (TIF) [file pone.0125648.s001.tif]

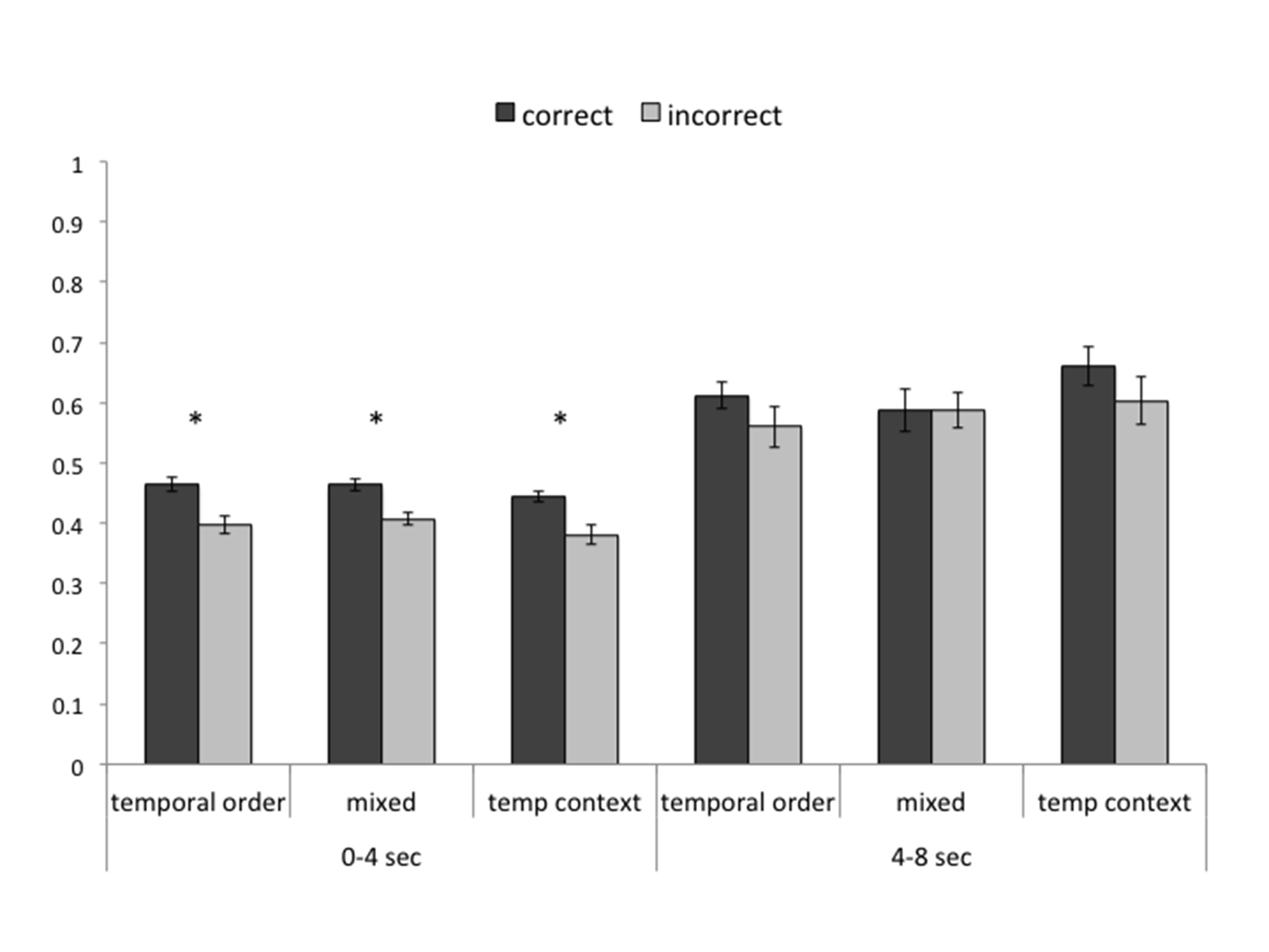

Supplement: S2 Fig — Error bars represent +/- standard error. * p < .05. (TIF) [file pone.0125648.s002.tif]
